# Assessing the Layer-by-Layer Assembly of Cellulose Nanofibrils and Polyelectrolytes in Pancreatic Tumor Spheroid Formation

**DOI:** 10.3390/biomedicines11113061

**Published:** 2023-11-15

**Authors:** Negar Abbasi Aval, Ekeram Lahchaichi, Oana Tudoran, Farzaneh Fayazbakhsh, Rainer Heuchel, Matthias Löhr, Torbjörn Pettersson, Aman Russom

**Affiliations:** 1 Division of Fibre Technology, Department of Fibre and Polymer Technology, KTH Royal Institute of Technology, 10044 Stockholm, Sweden; 2 Science for Life Laboratory, Division of Nanobiotechnology, Department of Protein Science, KTH Royal Institute of Technology, 17165 Solna, Sweden; 3 Department of Genetics, Genomics and Experimental Pathology, The Oncology Institute “Prof. Dr. I. Chiricuta”, 400015 Cluj-Napoca, Romania; 4 Pancreas Cancer Research Lab, Department of Clinical Science, Intervention and Technology, (CLINTEC), Karolinska Institutet, 17177 Stockholm, Sweden

**Keywords:** pancreatic ductal adenocarcinoma, three-dimensional tumor model, layer-by-layer, cellulose nanofibrils

## Abstract

Three-dimensional (3D) tumor spheroids are regarded as promising models for utilization as preclinical assessments of chemo-sensitivity. However, the creation of these tumor spheroids presents challenges, given that not all tumor cell lines are able to form consistent and regular spheroids. In this context, we have developed a novel layer-by-layer coating of cellulose nanofibril–polyelectrolyte bilayers for the generation of spheroids. This technique builds bilayers of cellulose nanofibrils and polyelectrolytes and is used here to coat two distinct 96-well plate types: nontreated/non-sterilized and Nunclon Delta. In this work, we optimized the protocol aimed at generating and characterizing spheroids on difficult-to-grow pancreatic tumor cell lines. Here, diverse parameters were explored, encompassing the bilayer count (five and ten) and multiple cell-seeding concentrations (10, 100, 200, 500, and 1000 cells per well), using four pancreatic tumor cell lines—KPCT, PANC-1, MiaPaCa-2, and CFPAC-I. The evaluation includes the quantification (number of spheroids, size, and morphology) and proliferation of the produced spheroids, as well as an assessment of their viability. Notably, our findings reveal a significant influence from both the number of bilayers and the plate type used on the successful formation of spheroids. The novel and simple layer-by-layer-based coating method has the potential to offer the large-scale production of spheroids across a spectrum of tumor cell lines.

## 1. Introduction

Spheroid-based three-dimensional (3D) cell cultures have, in recent years, gained significant attraction over the use of two-dimensional (2D) cell cultures. In 2D cultures, the cells are cultured as monolayers, and the interactions between cells and their surrounding extracellular matrix are diminished, leading to a restricted level of cellular responsiveness [1]. Unlike this artificial 2D culturing setup, cells in the human body exist within a 3D environment, and this spatial dimension is vital for their proper metabolism and growth [2,3]. By utilizing 3D cell cultures, researchers can effectively explore various aspects, including cancer cell behavior, intracellular interactions, and cell differentiation. Moreover, 3D cultures serve as improved/better tools for assessing substance toxicity and gauging the effectiveness of potential drugs, bridging the gap between 2D culturing and animal experiments [4]. Notably, 3D spheroids, which mimic solid avascular tumor nests, feature gradients for nutrients, oxygen, and waste products as well as permeability barriers that challenge the penetration of certain substances or test agents, adding a realistic dimension to investigations [5,6]. Consequently, spheroids, often termed “microtissues”, have become increasingly popular in the study of fundamental biology or for tissue engineering applications [7]. 

However, there are several obstacles related to utilizing spheroid models as 3D tumor models and assessing their in vivo effectiveness, such as generating and maintaining uniform spheroids, both in size and shape, making spheroids with minimal cell counts, and fabricating in vivo tumor models composed of diverse cell varieties [8]. Various traditional methods for generating 3D spheroids exist, including hanging drop [6,7], rotary cell culture systems [9], magnetic levitation [10], low-binding well plates [11], and hydrogel-based cultures [12]. However, each method comes with its own set of advantages and limitations. The use of low binding surfaces, which prevents cell adhesion to the material’s surface during cell culturing, emphasizes cell–cell interactions rather than interactions with the culture surface. Alternatively, a 3D cell culture can be achieved by seeding cells in artificial matrices, such as solid scaffolds (sponges or foams), hydrogels, fibers, or beads [13]. These matrices can be engineered with varying porosities and mechanical properties to mimic the in vivo tumor microenvironment. Non-adhesive polymers like agarose and hyaluronic acid (HA), either alone or blended with other biomaterials, are commonly used to create these scaffold-based materials, as their chemical and structural properties can be optimized to suit specific requirements [14,15].

Cellulose and its derivatives, such as sodium carboxymethyl cellulose (CMC) and the crowding agent methylcellulose, are highly valuable as semi-solidification agents in 3D cultures. By incorporating these compounds, a stable and supportive matrix can be created for nurturing cellular growth and facilitating tissue development [16]. The effectiveness of gelatin–carboxymethyl cellulose (G-CMC) hydrogels in supporting the growth of cancer spheroids has been demonstrated [17]. These spheroids, cultivated within the G-CMC matrix, exhibited comparable timelines and growth patterns to those grown in other matrices containing collagen, gelatin, or Matrigel. Remarkably, the viability of the spheroids remained excellent, even as hypoxia levels increased [17]. Considering the potential significance of CMC in spheroid formation as a 3D tumor model, we devised a layer-by-layer (LbL) technique utilizing polyelectrolytes and carboxymethylated cellulose nanofibrils (CNFs) to create a coating on well plate surfaces [18]. This innovative approach proved successful in promoting spheroid formation. Even with a low initial cell-seeding concentration, we were able to achieve spheroid generation on these LbL-CNF-coated surfaces within 24 h, without compromising cell viability. Furthermore, we confirmed the response of these spheroids generated from the HCT 116 cell line to an anti-cancer drug. In the current work, we further optimized the novel LbL-based nanofilm coating methods and applied them to grow spheroids of pancreatic tumor cell lines. 

Pancreatic ductal adenocarcinoma (PDAC) ranks among the top five deadliest cancer types in the Western world [19]; progress in treatment developments is being hindered by its complex physiopathology. PDAC is characterized by a heterogeneous cellular composition, and a significant challenge in this field is the lack of appropriate PDAC in vitro models that are capable of accurately replicating the tumor microenvironment for effective preclinical drug screening. Recognizing the critical need for improved PDAC models, this paper presents an improved protocol/technique for generating PDAC 3D cultures. Building on our previously published LbL-CNF coating platform [18], by increasing the number of bilayers to 10 and utilizing higher-molecular-weight polyelectrolytes on non-treated surfaces, we successfully developed a versatile platform capable of generating spheroids from several PDAC cell lines, even when starting with a low number of cells.

The objectivity of this paper is to (i) introduce a novel platform for creating spheroids using PDAC tumor cells by modifying the LbL-CNF coating, (ii) evaluate the effect of the molecular weight of the used polyelectrolytes, (iii) determine the optimal number of bilayers on two different types of well plates, and (iv) find how the number of seeded cells control the size and morphology of the formed spheroids.

## 2. Materials and Methods

### 2.1. Materials

The PANC-1, MiaPaCa-2, and CFPAC-I pancreatic cancer cell lines were acquired from the American Type Culture Collection (ATCC). The KPCT cells were a kind donation from R. Heuchel. The following reagents were purchased from Sigma-Aldrich, Germany: Dulbecco’s modified Eagle’s medium-F12 (DMEM-F12), DMEM high glucose, Roswell Park Memorial Institute (RPMI) medium, Fetal Bovine Serum (FBS), Penicillin–Streptomycin, Trypan Blue solution 0.4% liquid sterile-filtered, and Trypsin EDTA solution 1×. Polyallylamine hydrochloride (PAH) (MW = 725,000 g mol^−1^ and 17,500 g mol^−1^) was obtained from Polyscience, Niles, IL, USA, while Polyethyleneimine (PEI) (MW = 60,000 g mol^−1^ and 25,000 g mol^−1^) was purchased from Sigma-Aldrich, Taufkirchen, Germany. Carboxymethylated cellulose nanofibrils (CNFs) with a charge density of 600 μeq g^−1^ were sourced from RISE, Sweden, and further prepared as described in [18]. Nuclear fluorescent stains, Hoechst, Calcein-AM, and Propidium Iodide (PI) were purchased from Thermo Fisher Scientific, Stockholm, Sweden. Tissue-culture-treated (TC) plates, Nunclon delta (Nunc) plates, and Non-treated-non sterilized (NT/NS) plates were purchased from Thermo Fisher Scientific, Stockholm, Sweden, and Ultra-Low Attachment (ULA) plates were purchased from Corning, Glendale, AZ, USA.

### 2.2. Layer-by-Layer Coating in 96-Well Plates

Nanometer-thin films of CNFs were built on 96-well plates through the addition of various numbers of PAH (CNF/PEI)_n_ layers, following the protocol described in [18] with modifications. The differences lie in the utilization of high-molecular-weight (HMW) polyelectrolytes instead of their LMW counterparts and increasing the number of bilayers from 5 to 10. Both the Nunc and NT/NS plates were tested for optimal surface selection. Briefly, the assembly started by adding 100 μL of a high-molecular-weight (HMW) cationic PAH solution (MW = 725,000 g mol^−1^) at a concentration of 100 mg L^−1^ in Milli-Q water (MQ) (pH 7.5) to coat the bottom of the wells. The plates were incubated at room temperature (RT) for 10 min to facilitate adsorption and subsequently washed three times with 100 μL of MQ water. Next, 100 μL of an anionic CNF solution (100 mg L^−1^), prepared in MQ water following the protocols outlined in [18], was added to the wells and incubated at RT for an additional 10 min. After the incubation period, the CNF solution was removed, and the wells underwent three washes with 100 μL of MQ water. A solution of HMW PEI (MW = 60,000 g mol^−1^) at a concentration of 100 mg L^−1^ in MQ water was subsequently added into the wells and allowed to adsorb for 10 min. This process resulted in the formation of PAH (CNF/PEI) layers. The process of adding CNF and PEI was repeated up to 5 and 10 bilayers of CNFs. The layering process was terminated when the outermost layer in all coated wells consisted of CNFs. Ultra-Low Attachment (ULA) plates (without any additional LbL-CNF coating) were used as a standard control method for spheroid formation.

### 2.3. Spheroid Formation

Cell suspensions ranging from 10 to 1000 cells in a 100 μL medium were seeded on 5 and 10 bilayers of CNF-coated TC, Nunc, and NT/NS 96-well plates, as well as the ULA control plate. Throughout the culturing period, the plates were placed in an incubator with 5% CO_2_ at 37 °C, and the medium was replaced every 2 days. The process of spheroid formation was monitored using a ZOE Fluorescent cell imager (Bio-RAD, model 1450031, Sweden), for a duration of 10 days, on days 3, 7, and 10. The size of the spheroids based on their diameter was measured manually using the ImageJ (1.52 a) software. 

### 2.4. Live–Dead Assay

The viability of the generated spheroids was assessed using a Live/Dead staining kit. Before staining, the growth medium was removed, and the spheroids were washed once with phosphate-buffered saline (PBS). Next, 100 μL of 1 mg mL^−1^ Calcein AM (520 Da), 1 mg mL^−1^ Propidium Iodide (PI) (668 Da), and 1 μg mL^−1^ Hoechst stain mixtures in PBS were added to the wells and the plates were incubated in the dark at 37 °C for 30 min. The stained cells were visualized using a fluorescent microscope (Nikon Eclipse Ti, Tokyo, Japan, paired with a Lumencor SOLA light, Beaverton, OR, USA, as an excitation source) and the resulting images were analyzed using the ImageJ software.

### 2.5. Statistical Analysis

The data are presented as mean values and standard deviation, and the statistical significance of group comparisons was assessed using the non-parametric Mann–Whitney U Test. A *p*-value of less than 0.1 was considered to be statistically significant.

## 3. Results and Discussion

### 3.1. PDAC Spheroid Formation on Different Plates

**Figure 1:**
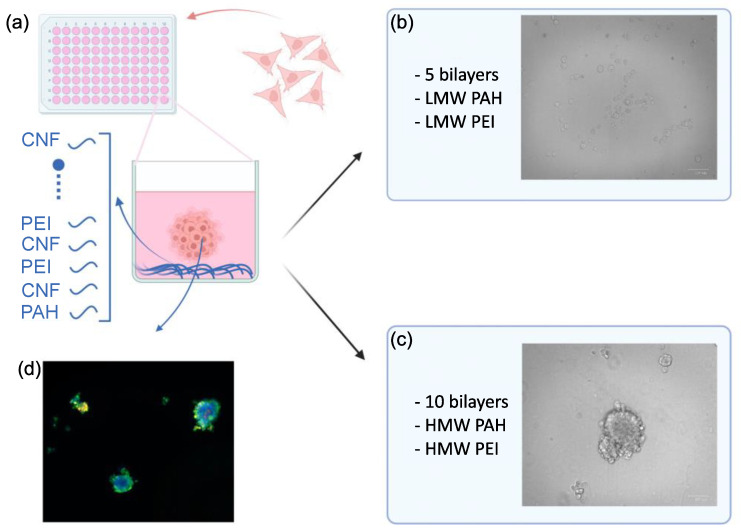
Schematic of PANC-1 spheroid formation on 5- and 10-bilayer assemblies of CNF and polyelectrolytes. (**a**) PANC-1 seeding on an LbL-CNF assembly-coated 96-well plate, (**b**) PANC-1 cells adhering in wells coated with 5 bilayers of CNF-polyelectrolyte, (**c**) spheroid formation of PANC-1 cells on wells coated with 10 bilayers of CNF-polyelectrolyte, and (**d**) a fluorescent image of the PANC-1 spheroids (nucleus in blue (Hoechst), green viable cells (Calcein), and red dead cells (PI stain)) on wells coated with 10 bilayers of CNF-polyelectrolyte.

A schematic depicting the coating process and PDAC spheroid generation is presented in Figure 1. Briefly, the plate is coated with multilayers (e.g., LbL) of CNF/polyelectrolytes according to our previously published method [18] followed by growing cells (Figure 1a). Initially, the TC wells were coated with five bilayers using low-molecular-weight polyelectrolytes (Figure 1b). However, as shown in Figure 1b, for the PANC-1 cells, the method did not generate spheroids. Similar results were obtained for the other PDAC cell lines tested (Supplementary Materials Figure S1). The KPCT cells exhibited complete expansion covering the entire surface, while the PANC-1, MiaPaCa-2, and CFPAC-I cells remained in a rounded single-cell shape, failing to aggregate and form spheroids. All in all, the method did not generate robust spheroids for the PDAC cell lines. We speculate that this could be attributed to the replacement of the LMW polyelectrolytes used in the LbL-CNF coating by HMW proteins in the cell culture media, potentially leading to a breakdown of the LbL coating [20].

We hypothesized that increasing the molecular weight of the polyelectrolytes and increasing the number of layers could facilitate PDAC 3D spheroid formation. Several protocol modifications were tested, revealing that an increase in the number of bilayers concomitant with the higher molecular weight of the polyelectrolytes enabled the formation of well-defined spheroids (Figure 1c,d). To test the effect of the molecular weight and increased number of layers more in detail, we tested different plates (Nunc, NT/NS) with either five or ten CNF-HMW polyelectrolyte bilayers at different cell-seeding concentrations (10 to 1000 cells per well).

A comprehensive overview of the characterization results is provided in Supplementary Materials Figures S2–S5. Overall, a trend of increasing the number of formed spheroids with higher cell-seeding concentrations is observed consistently across both five and ten CNF-polyelectrolyte bilayer-covered plates. This suggests that opting for a higher seeding concentration can be advantageous, leading to the production of an increasing number of spheroids within otherwise identical culture conditions. This observation aligns well with prior research, which has demonstrated that 3D tumor spheroid cultures within microwell plates often necessitate markedly elevated cell-seeding concentrations [18,21]. Furthermore, the NT/NS plates coated with 10 bilayers of CNF-HMW polyelectrolytes showed the most favorable outcome, promoting the spontaneous formation of cell spheroids. 

**Figure 2:**
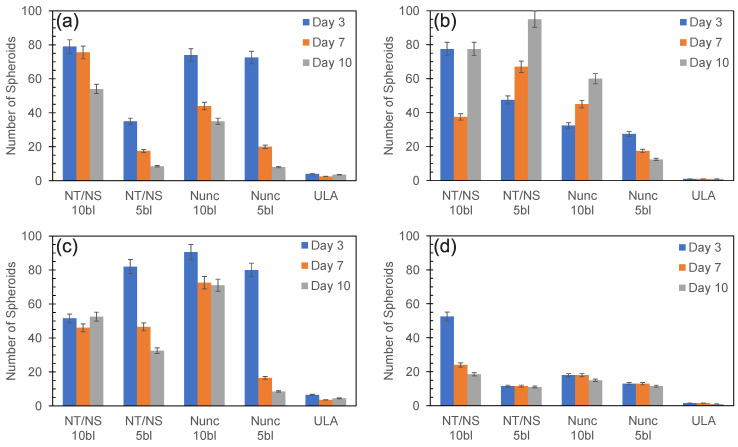
The number of spheroids formed (counted 3, 7, and 10 days after seeding) on different plates covered with 5 and 10 CNF-HMW polyelectrolyte bilayers of 500 cells from (**a**) KPCT, (**b**) PANC-1, (**c**) MiaPaCa-2, and (**d**) CFPAC-I cell lines.

Figure 2 shows the number of spheroids stemming from the seeding and culture of 500 cells across all four cell lines, for all plate types counted on days 3, 7, and 10. As can be seen, both plates (NT/NS and Nunc) coated with five and ten bilayers were able to form spheroids across all PDAC cell lines tested. Except for PANC-1 cultured in NT/NS plates (see Figure 2b), after 10 days of culture, the cell lines generated more spheroids for the ten bilayers compared to the five bilayers in both plates. Furthermore, in most culture conditions, there is a decline in the number of spheroids over time (see days 3 and 10 in Figure 2), presumably due to the merging of the formed spheroids, resulting in a reduction in the number of spheroids. However, for the PANC-1 and MiaPaCa-2 cell lines cultured in the ten-bilayer NT/NS plates (Figure 2b,c), there is a slight decrease in spheroid count from day 3 to day 7 followed by an increase from day 7 to day 10. This phenomenon is potentially driven by an increased proliferation rate compared to the other cell lines (KPCT and CFPAC-I) [22,23]. 

Across all the plates tested (NT/NS and Nunc, coated with five and ten bilayers), under identical culture conditions and with equivalent cell concentrations, the tumor spheroid formation resulted in a lower number of formed spheroids on the ULA plates (Supplementary Materials Figures S2–S5). These cumulative findings collectively underscore the inherent property of the CNF-polyelectrolyte LbL coating approach in enabling high-throughput spheroid formation using a standard culture setup. The formation of spheroids from a limited number of primary cells holds significant importance, particularly in the field of personalized medicine and patient-specific treatments. Given the constraints of working with low cell-seeding concentrations in these contexts, the ability to generate spheroids from such limited cell numbers is promising [24,25].

### 3.2. Spheroid Morphology and Size

**Figure 3:**
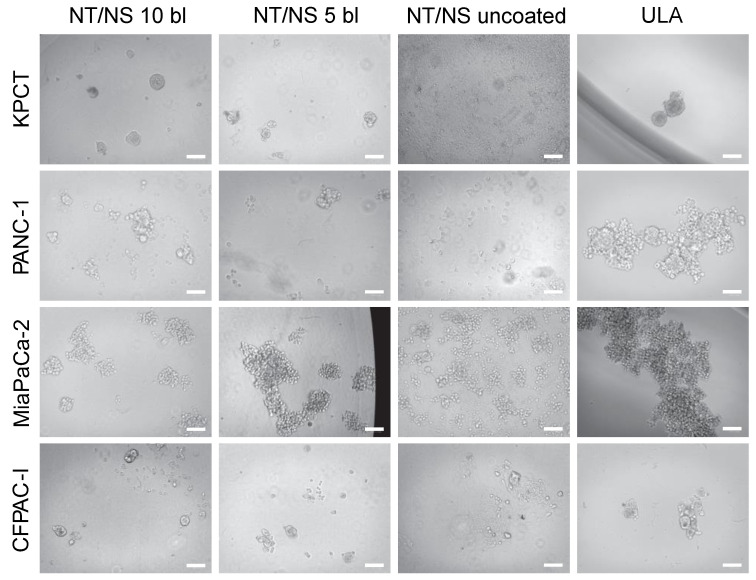
Spheroids formed of 500 cells from different cell lines (KPCT, PANC-1, MiaPaCa-2, and CFPAC-1) on NT/NS plate coated with 10 bilayers, NT/NS plate coated with 5 bilayers, uncoated NT/NS plate, and ULA plate (scale bar = 100 μm).

To examine the growth of spheroids generated using different plates, the morphology and diameter of the spheroids were analyzed. The morphology of spheroids, cultivated with 500 cells for 7 days in NT/NS plates, is illustrated in Figure 3, and the morphology of spheroids formed in Nunc plates is depicted in Supplementary Materials Figure S3. As can be seen in Figure 3, successful spheroid formation is observed for the KPCT cells on the NT/NS plates with five- and ten-bilayer coatings, and the resulting morphology is circular, closely resembling the spheroids formed on the ULA plate. However, for these cells, spheroid formation is impeded on the Nunc plates with both five- and ten-bilayer coatings. For the PANC-1 cell line, spheroids are formed on NT/NS plates with five- and ten-bilayer coatings, but their structure appears irregular, and more resemble aggregation. However, the morphology aligns with cells cultured on the ULA plate. Spheroid formation fails for the PANC-1 on the Nunc plate with a five-bilayer coating, whereas successful spheroid formation could be seen on the Nunc plate with a 10-bilayer coating. It is worth highlighting that spheroid formation failed for the Nunc plate with 10-bilayer coatings for the tested cell lines, where some cells remain in a single-cell state (Supplementary Materials Figure S6). For the MiaPaCa-2 cells, successful spheroid formation is observed across all plates with both five- and ten-bilayer coatings (both for NT/NS and Nunc plates). Notably, the morphology of spheroids formed on the NT/NS plate with a 10-bilayer coating resembles the spheroids formed on the ULA plate, characterized by substantial aggregations. Conversely, spheroids developed on the other plates (NT/NS 5 bl, Nunc 10 bl, and Nunc 5 bl) exhibit smaller sizes. Finally, the CFPAC-I cells demonstrate a spheroid formation capability on the NT/NS plates with both five- and ten-bilayer coatings (Figure 3), but not on the Nunc plate with a 10-bilayer coating (Supplementary Materials Figure S6). However, in the presence of a five-bilayer coating on the Nunc plate, a few spheroids can be observed (Supplementary Materials Figure S6). As a control, 2D adhesion and spreading of cells from the four cell lines onto uncoated NT/NS plates is observed, indicating that the pivotal determinant lies within the LbL coating, which could govern the cells’ capacity to undergo spheroid formation.

Next, we evaluated the spheroid proliferation by examining the size arising from the seeding of the cell lines within the NT/NS plate, coated with a 10-bilayer coating (Supplementary Materials Figure S7). We observed increased spheroid sizes as time progressed for all cell lines and seeding concentrations (10, 100, 200, 500, or 1000 cells/well). Examining the progression of the spheroid, it becomes evident that all cell lines cultivated on the NT/NS plate coated with 10 bilayers exhibited an increasing size growth over time. This suggests that the spheroids formed within this context result from a combination of cellular aggregation and concurrent proliferation. While more experiments are needed to fully study this, it is indicative that the platform can offer additional benefits to the current models [11], where spheroid formation is considered to be primarily attributed to aggregation alone. Hence, we speculate that this platform has promising potential to generate spheroids that closely emulate in vivo tumors compared to aggregation-based methods. 

**Figure 4:**
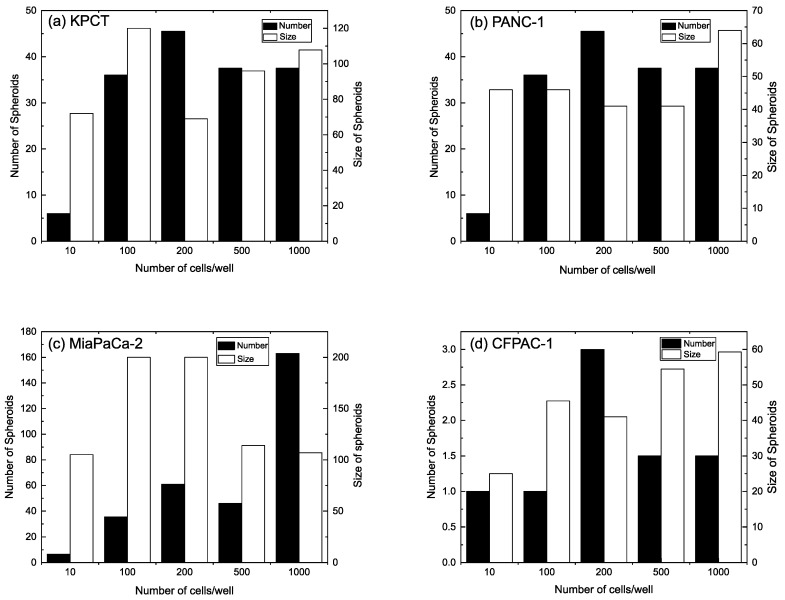
Size and number of spheroids formed of different cell lines, (**a**) KPCT, (**b**), MiaPaCa-2, (**c**) PANC-1, and (**d**) CFPAC-I in different concentrations (10, 100, 200, 500, and 1000 cells/well) in NT/NS plates covered with 10 bl after 7 days.

When comparing the size and number of spheroids formed in the NT/NS plates coated with a 10-bilayer assembly of CNF and HMW polyelectrolytes (Figure 4), several observations can be made. First, the spheroid formation predominantly results from cell proliferation rather than from cell aggregation. A closer examination of Figure 4 highlights a consistent trend, in which both the size and number of spheroids increase simultaneously with the increasing amount of initial seeding. These observations further confirm our hypothesis regarding spheroid formation through a combination of proliferation and aggregation. Furthermore, with the growing number of spheroids, there is a concurrent increase in the size of individual spheroids. This size increment is not solely attributable to the aggregation of spheroids but also the proliferation of the constituent cells within each spheroid. As mentioned previously, MiaPaCa-2 exhibits a higher proliferation rate compared to the other cell lines [22]. 

Consequently, newly formed spheroids experience rapid internal cell proliferation and growth before aggregating with others. This behavior results in a decrease in the size of individual spheroids as the overall number of spheroids increases. Furthermore, for the CFPAC-I cell line, the number of spheroids is reduced while their size increases. This phenomenon might be attributed to the relatively lower proliferation rate of CFPAC-I cells in comparison to other cell lines. The slow proliferation rate could lead to the spheroids merging together before the cells have the opportunity to proliferate further.

### 3.3. Analysis of Spheroids’ Viability

**Figure 5:**
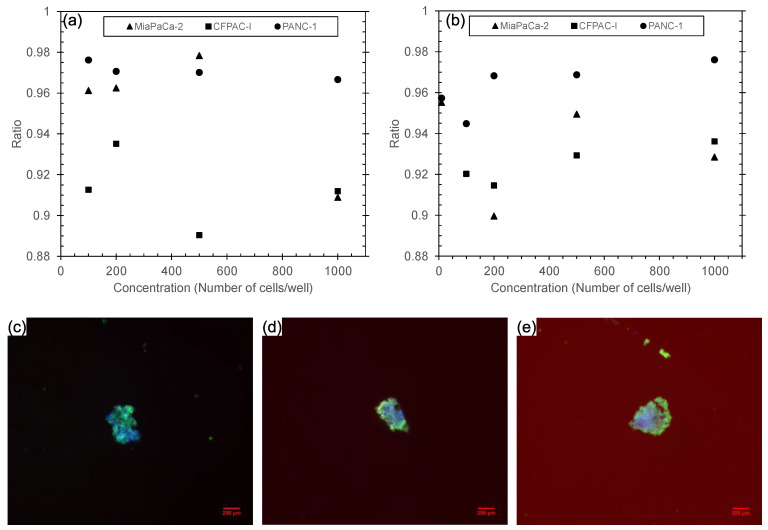
Total alive ratio from cell lines PANC-1, MiaPaCa-2, and CFPAC-1 on day 7 in (**a**) NT/NS plate coated with 10 CNF-HMW bilayers and (**b**) ULA plate. (**c**–**e**) Images of live–dead cells in PANC-1 cell lines with (**c**) 100, (**d**) 200, and (**e**) 500 cells/well on day 7 in NT/NS plate coated with 10 CNF-HMW bilayers (scale bar = 200 μm).

A viability assessment was conducted on spheroids cultivated within wells coated with CNF-polyelectrolyte bilayers for three pancreatic cell line variations (PANC-1, MiaPaCa-2, and CFPAC-I). The staining experiments and fluorescence intensity measurements were performed for viable and apoptotic cells in two distinct settings: the NT/NS plate coated with 10 bilayers (Figure 5a) and the ULA plate (Figure 5b). In all cell lines and on both types of plates, the majority of cells were found to be alive after 7 days of culture. This can be seen by the ratio between the live and dead cells (Figure 5a,b), which was between 90 and 98% across the cell lines and plates. The images of fluorescently labeled spheroids derived from 100, 200, and 500 PANC-1 cells are presented in Figure 5c–e. As expected, the majority of cells within the spheroids, both internally and along the periphery, exhibit viability, as indicated by their green fluorescence.

The homocellular spheroids comprising PDAC, established on the LbL-CNF platform, hold potential applications in various fields including tumor drug studies, tumor-on-a-chip technology, and personalized medicine through the utilization of primary cells extracted from the body.

## 4. Conclusions

Recently, we introduced an innovative coating method involving CNF-polyelectrolyte bilayers for the generation of spheroids [15]. In this present work, we have further enhanced the CNF-polyelectrolyte LbL coating technique and applied the optimized protocol for pancreatic ductal adenocarcinoma cells. We demonstrated the capacity for spheroid formation and subsequent proliferation across four pancreatic ductal adenocarcinoma cell lines—PANC-1, MiaPaCa-2, KPCT, and CFPAC-I. Spheroid formation for PDAC is particularly significant due to the persistent prominence of pancreatic ductal adenocarcinoma as a major contributor to mortality among solid tumors [15]. In part, the choice to focus on PDAC is due to the absence of suitable preclinical models to effectively address this challenge. Furthermore, this analysis encompasses multiple facets, including the quantification of spheroid counts on four different surfaces (NT/NS and Nunc) and different numbers of coated bilayers (five and ten). It also involves the exploration of spheroid morphology across these same surfaces, tracking spheroid growth dynamics over time, and verifying the biocompatibility of our LbL coating through the assessment of spheroid viability. To apply spheroids in PDAC, enhancements are essential to develop co-culturing models with stromal cells or epithelial cells that form 3D models for investigating the interaction between cancer cells and elements within the ECM [26].

## Data Availability

Data are available upon request from the corresponding authors.

## Supplementary Materials

The following supporting information can be downloaded at https://www.mdpi.com/article/10.3390/biomedicines11113061/s1. Figure S1: The morphology of cells (KPCT, PANC-1, MiaPaCa-2 and CPFAC-1) on TC plates covered with 5 CNF-LMW polyelectrolyte bilayers of different cell concentrations (10, 100, 200, 500 and 1000 cells/well) after 7 days. (Scale Bar = 100 μm); Figure S2: Number of spheroids in different plates (NT/NS 10 bl, NT/NS 5 bl, Nunc 10 bl, Nunc 5 bl and ULA) for (a) 10, (b) 100, (c) 200, (d) 500 and (e) 1000 KPCT cells/well counted after 3, 7, 10 days; Figure S3: Number of spheroids in different plates (NT/NS 10 bl, NT/NS 5 bl, Nunc 10 bl, Nunc 5 bl and ULA) for (a) 10, (b) 100, (c) 200, (d) 500 and (e) 1000 PANC-1 cells/well counted after 3, 7, 10 days; Figure S4: Number of spheroids in different plates (NT/NS 10 bl, NT/NS 5 bl, Nunc 10 bl, Nunc 5 bl and ULA) for (a) 10, (b) 100, (c) 200, (d) 500, and (e) 1000 MiaPaCa-2 cells/well counted after 3, 7, 10 days; Figure S5: Number of spheroids in different plates (NT/NS 10 bl, NT/NS 5 bl, Nunc 10 bl, Nunc 5 bl and ULA) for (a) 10, (b) 100, (c) 200, (d) 500, and (e) 1000 CFPAC-I cells/well counted after 3, 7, 10 days; Figure S6: Spheroids formed of different cell lines (KPCT, PANC-1, MiaPaCa-2 and CFPAC-1) using 500 cells/wells in 100 μl media on a Nunc plate coated with 10 bilayers, 5 bilayers, and an uncoated Nunc plate (scale bar = 100 μm); Figure S7: Size of spheroids formed of different cell lines in different concentrations (10, 100, 200, 500, and 1000 cells/well) in NT/NS plate covered with 10 bl after 3, 7 and 10 days. Size of spheroids from (a) KPCT, (b), PANC-1, (c) MiaPaCa-2, and (d) CFPAC-I. For statistic evaluation of the size increase for 500 cells/well see Table S1; Table S1: Mann-Whitney-U-Test of the spheroid size comparing day 3 to day 10 for all cell lines (KPCT, PANC-1, MiaPaCa-2 and CFPAC-1) for the cell seeding number of 500 cells/well.
