# Supplementary material for: Assessing the Layer-by-Layer Assembly of Cellulose Nanofibrils and Polyelectrolytes in Pancreatic Tumor Spheroid Formation
Source: Biomedicines. 2023 Nov 15;11(11):3061. doi: 10.3390/biomedicines11113061 (PMC10669291; doi:10.3390/biomedicines11113061)
Supplement: Supplementary file 1 [file biomedicines-11-03061-s001.zip › biomedicines-2693743-supplementary.pdf]

Supplementary Information

Assessing the Layer-by-Layer Assembly of Cellulose Nanofibrils and Polyelectrolytes in Pancreatic Tumor Spheroid Formation

Negar Abbasi Aval, Ekeram Lahchaichi, Oana Tudoran, Farzaneh Fayazbakhsh, Rainer Heuchel, Matthias Löhr, Torbjörn Pettersson and Aman Russom

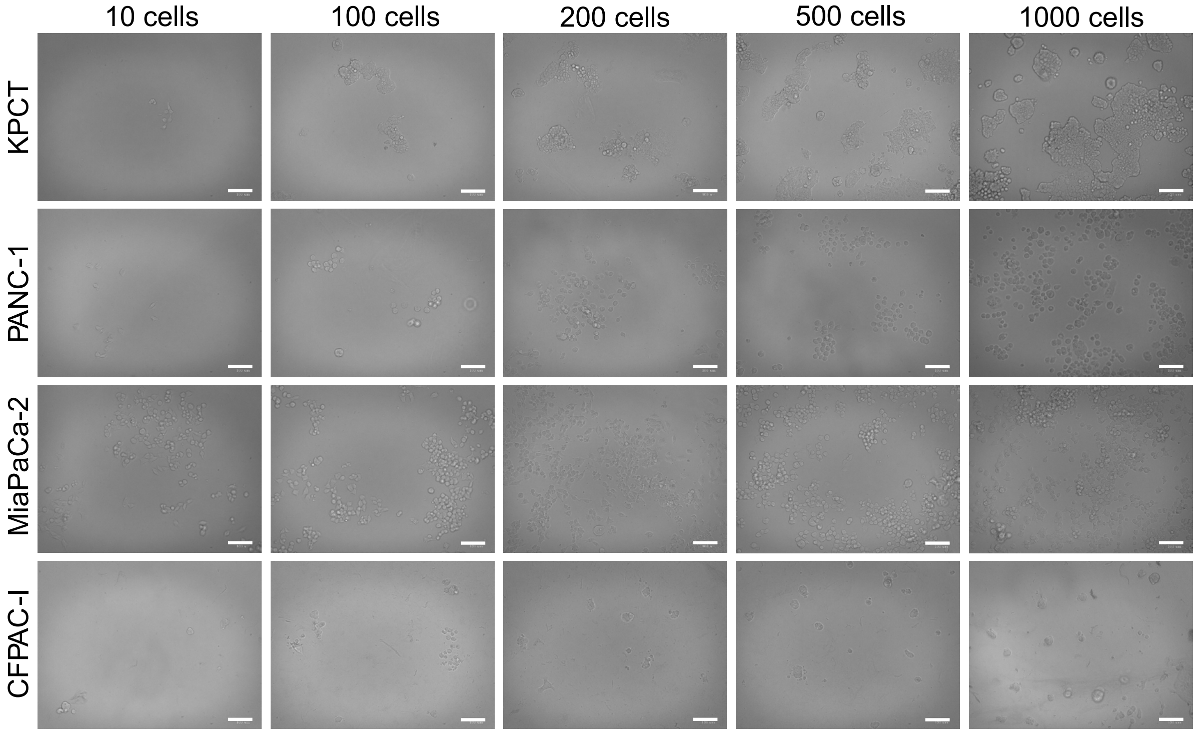

Figure S1. The morphology of cells (KPCT, PANC-1, MiaPaCa-2 and CPFAC-1) on TC plates covered with 5 CNF-LMW polyelectrolyte bilayers of different cell concentrations (10, 100, 200, 500 and 1000 cells/well) after 7 days. (Scale Bar=100 µm).

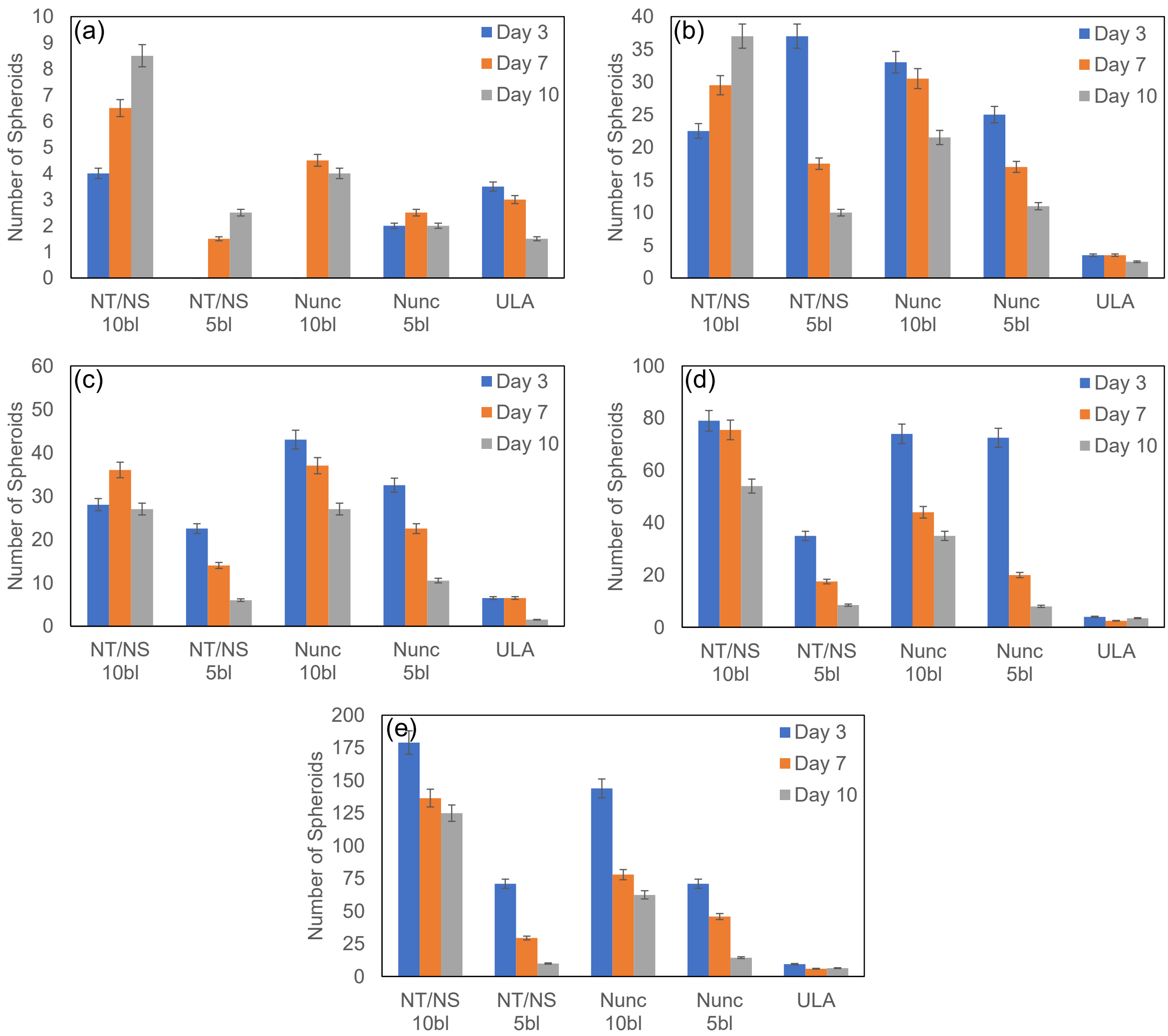

Figure S2. Number of spheroids in different plates (NT/NS 10bl, NT/NS 5bl, Nunc 10bl, Nunc 5bl and ULA) for (a) 10, (b) 100, (c) 200, (d) 500 and (e) 1000 KPCT cells/well counted after 3, 7, 10 days.

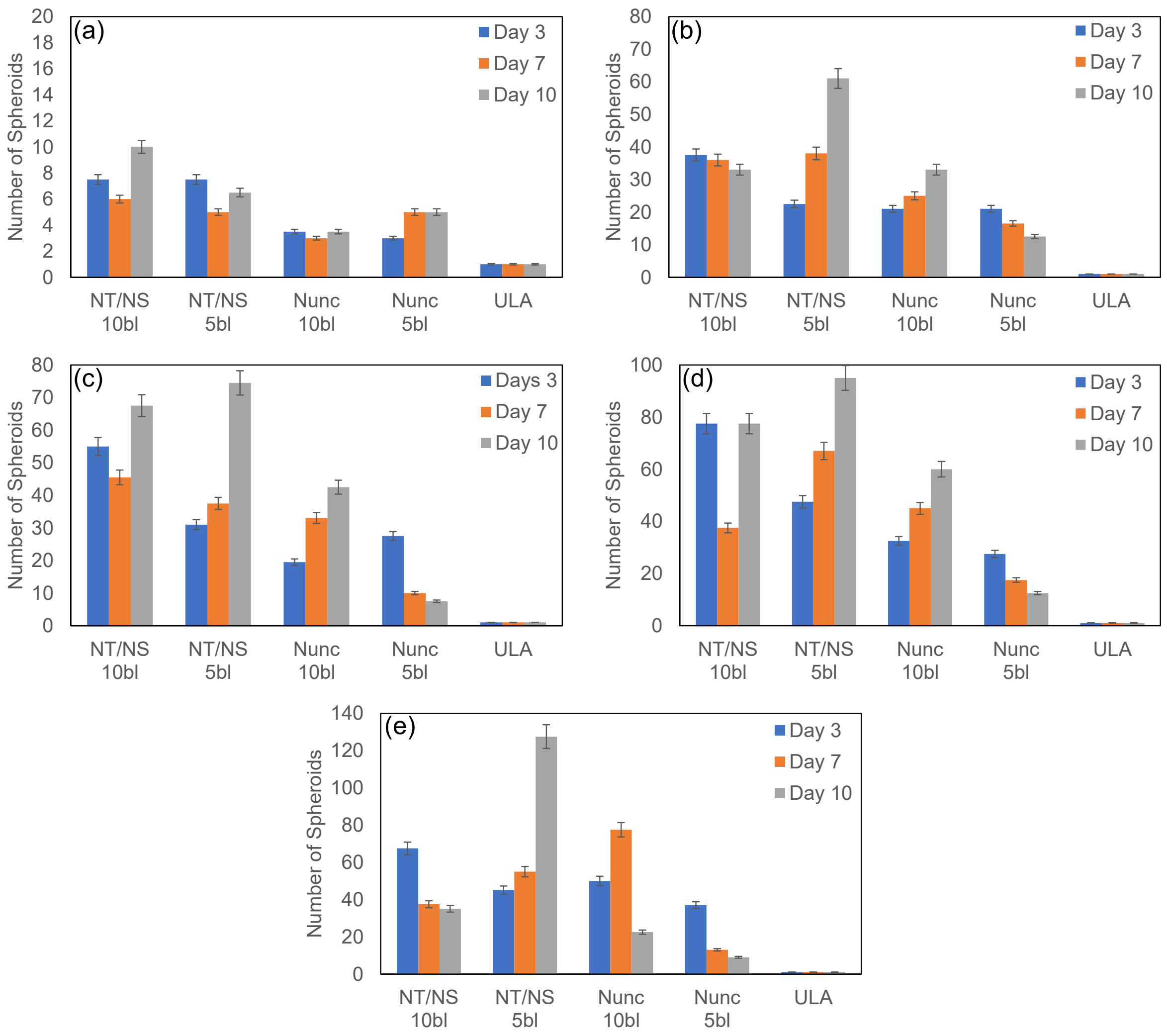

Figure S3. Number of spheroids in different plates (NT/NS 10bl, NT/NS 5bl, Nunc 10bl, Nunc 5bl and ULA) for (a) 10, (b) 100, (c) 200, (d) 500 and (e) 1000 PANC-1 cells/well counted after 3, 7, 10 days.

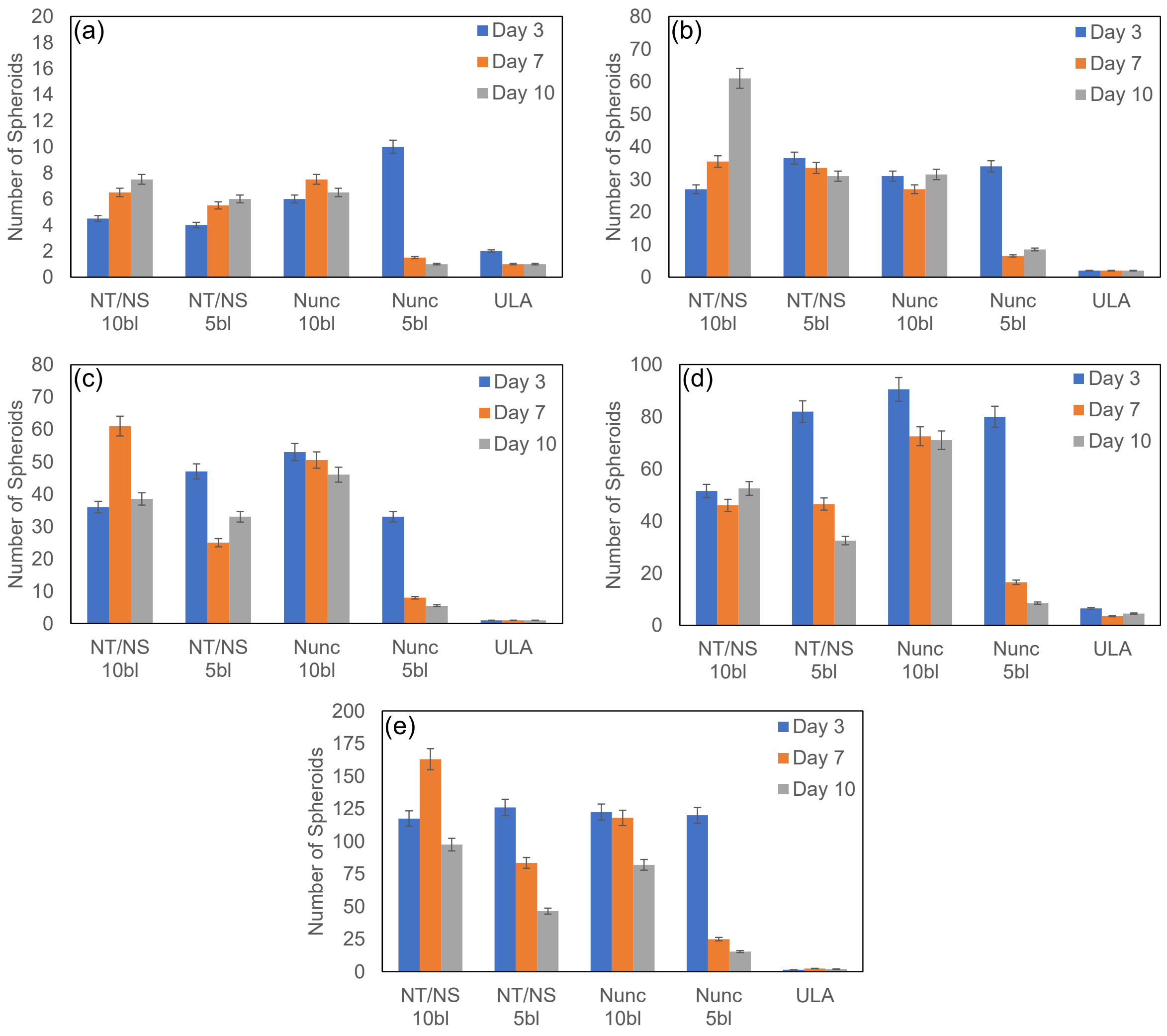

Figure S4. Number of spheroids in different plates (NT/NS 10bl, NT/NS 5bl, Nunc 10bl, Nunc 5bl and ULA) for (a) 10, (b) 100, (c) 200, (d) 500, and (e) 1000 MiaPaCa-2 cells/well counted after 3, 7, 10 days.

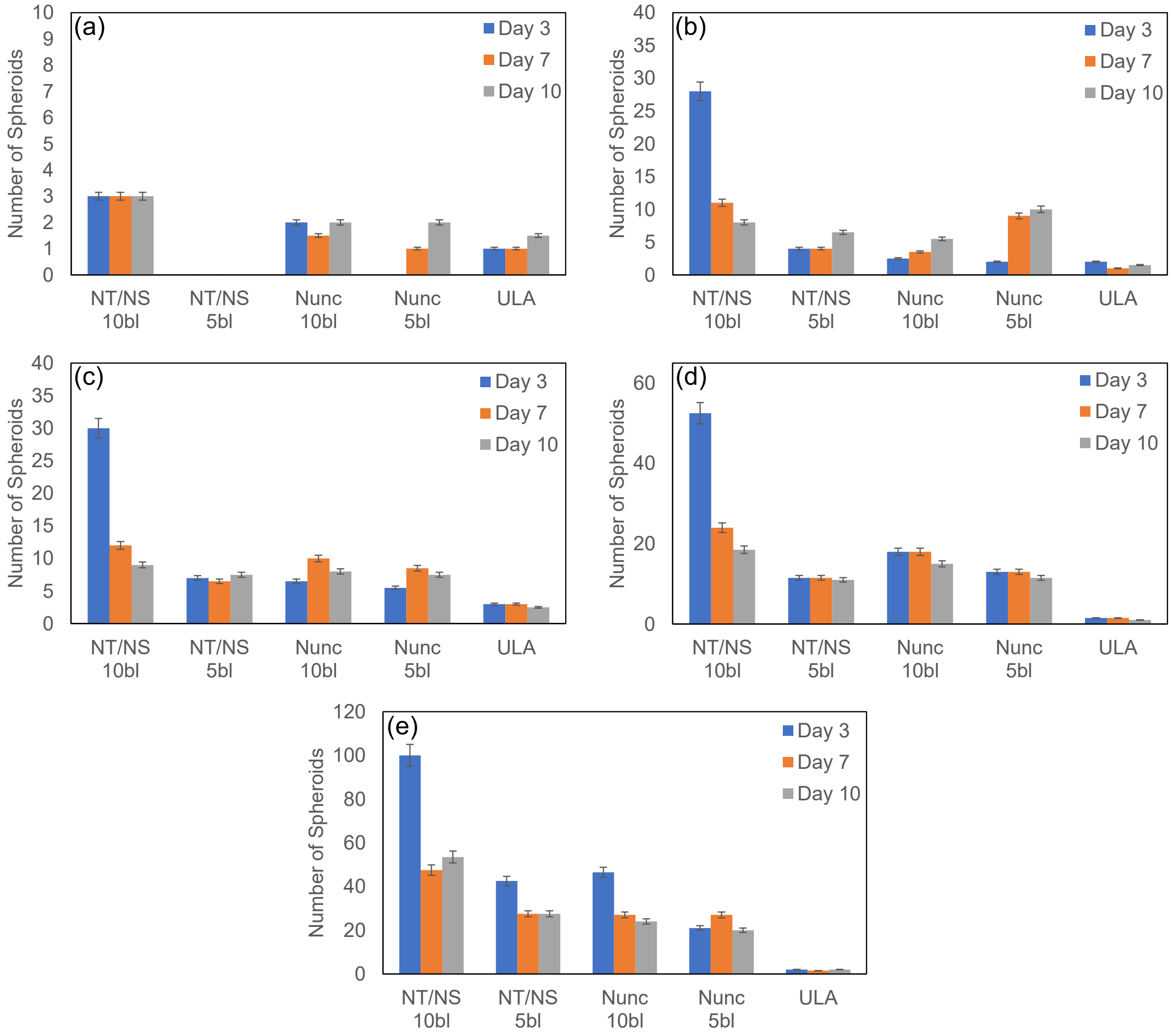

Figure S5. Number of spheroids in different plates (NT/NS 10bl, NT/NS 5bl, Nunc 10bl, Nunc 5bl and ULA) for (a) 10, (b) 100, (c) 200, (d) 500, and (e) 1000 CFPAC-I cells/well counted after 3, 7, 10 days.

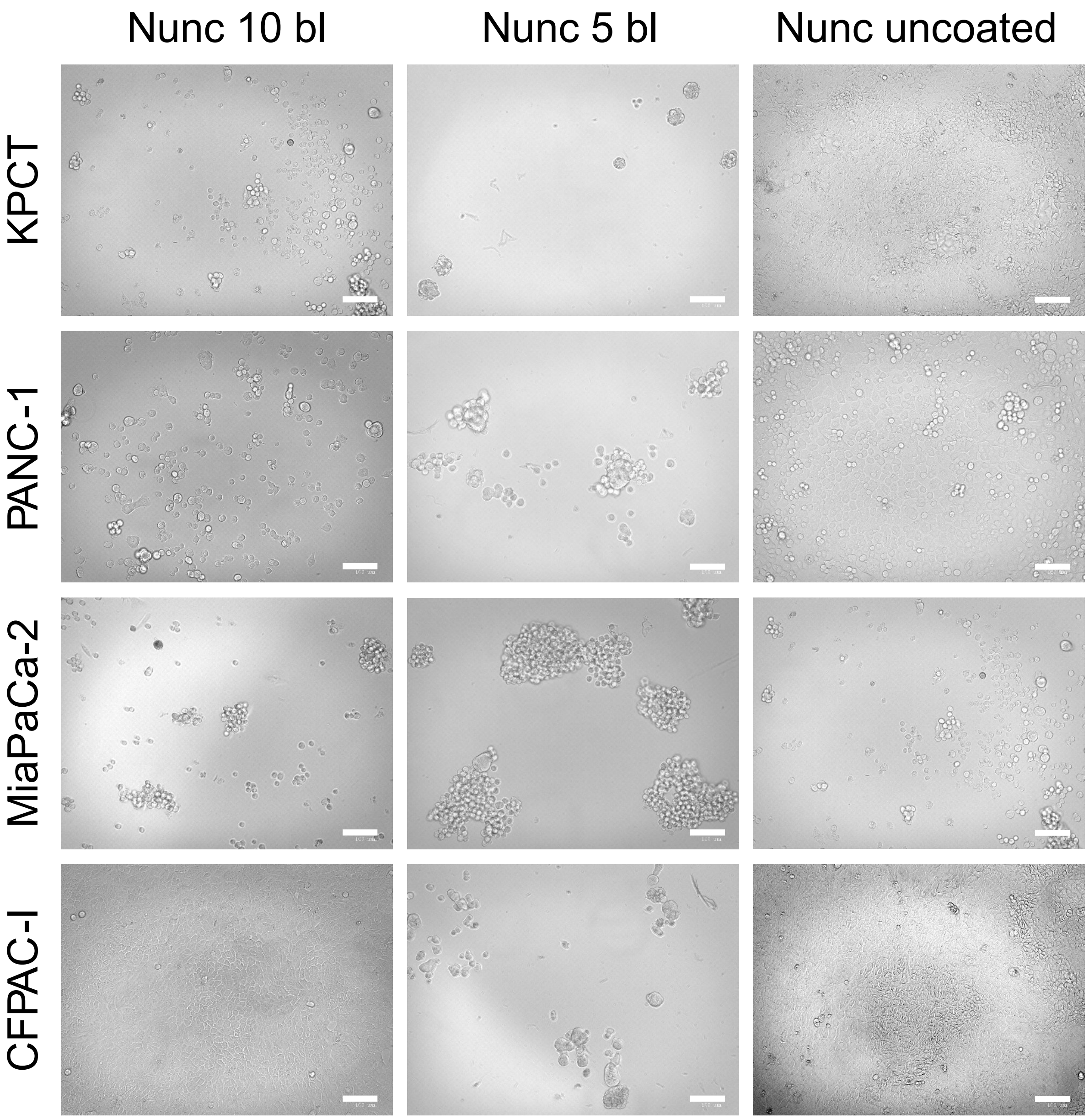

Figure S6. Spheroids formed of different cell lines (KPCT, PANC-1, MiaPaCa-2 and CFPAC-1) using500 cells/wells in 100 μl media on a Nunc plate coated with 10 bilayers, 5 bilayers, and an uncoated Nunc plate (scale bar=100 μm).

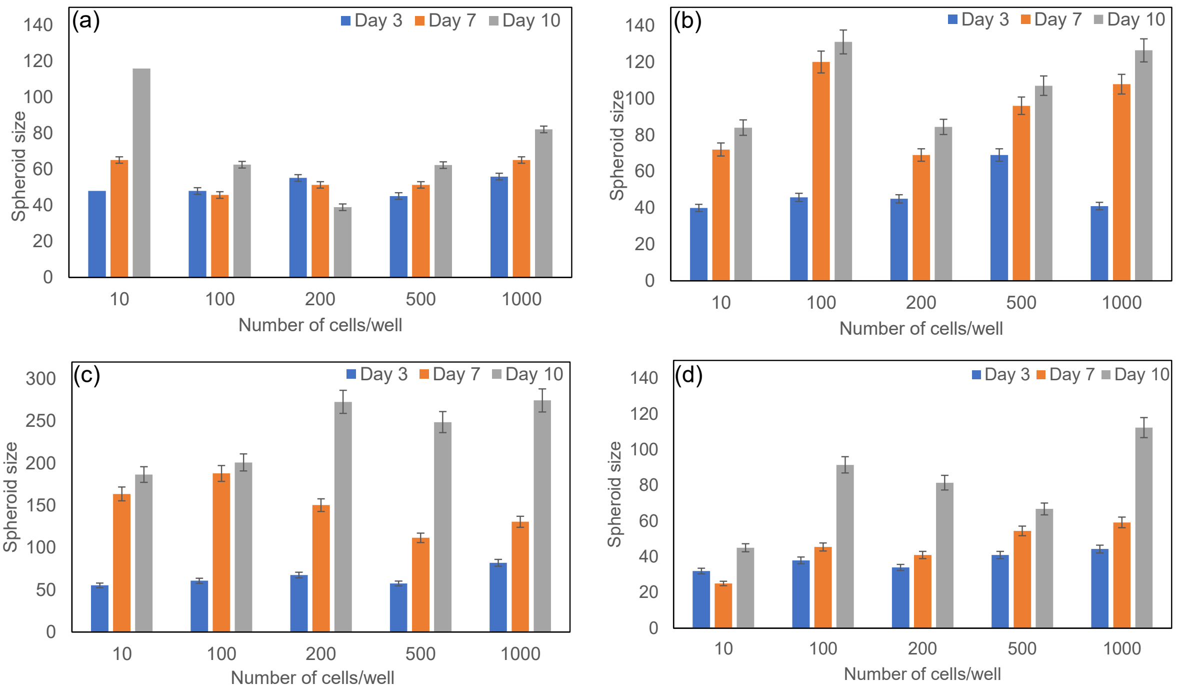

Figure S7. Size of spheroids formed of different cell lines in different concentrations (10, 100, 200, 500, and 1000 cells/well) in NT/NS plate covered with 10 bl after 3, 7 and 10 days. Size of spheroids from (a) KPCT, (b), PANC-1, (c) MiaPaCa-2, and (d) CFPAC-I. For statistic evaluation of the size increase for 500 cells/well see Table S1.

Table S1. Mann-Whitney-U-Test of the spheroid size comparing day 3 to day 10 for all cell lines (KPCT, PANC-1, MiaPaCa-2 and CFPAC-1) for the cell seeding number of 500 cells/well.

	p-value	U-value
KPCT	0.047	2.5
PANC-1	0.031	1
MiaPaCa-2	0.057	0
CFPAC-1	0.035	0
